# Supplementary material for: Mechanisms governing the pioneering and redistribution capabilities of the non-classical pioneer PU.1
Source: Nat Commun. 2020 Jan 21;11:402. doi: 10.1038/s41467-019-13960-2 (PMC6972792; doi:10.1038/s41467-019-13960-2)
Supplement: Supplementary file 7 — Source data [file 41467_2019_13960_MOESM7_ESM.zip › Source_Data/FigureS2/FigureS2B_MotifScanOutput/KmeansCluster2/homerResults/motif9.similar.html]

motif9

## Information for motif9

C
G
T
A
A
T
C
G
G
C
A
T
A
G
C
T
G
A
T
C
C
G
A
T
A
T
C
G
A
G
T
C
C
G
A
T
G
C
A
T
A
G
C
T
G
A
C
T
  
Reverse Opposite:  

C
T
G
A
T
C
G
A
C
G
T
A
G
C
T
A
T
C
A
G
T
A
G
C
C
G
T
A
C
T
A
G
T
C
G
A
C
G
T
A
T
A
G
C
G
C
A
T
  

|  |  |
| --- | --- |
| p-value: | 1e-105 |
| log p-value: | -2.441e+02 |
| Information Content per bp: | 1.686 |
| Number of Target Sequences with motif | 436.0 |
| Percentage of Target Sequences with motif | 8.68% |
| Number of Background Sequences with motif | 875.1 |
| Percentage of Background Sequences with motif | 2.15% |
| Average Position of motif in Targets | 104.5 +/- 50.2bp |
| Average Position of motif in Background | 98.2 +/- 70.8bp |
| Strand Bias (log2 ratio + to - strand density) | -0.0 |
| Multiplicity (# of sites on avg that occur together) | 1.02 |
| Motif File: | file (matrix) reverse opposite |

### Similar de novo motifs found

|  |  |  |  |  |  |  |  |
| --- | --- | --- | --- | --- | --- | --- | --- |
| Rank | Match Score | Redundant Motif | P-value | log P-value | % of Targets | % of Background | Motif file |
| 1 | 0.905 | C G T A A T G C A C G T A C G T A G T C C G A T A C T G A G T C C G A T G C A T G A C T G A C T C G A T A C T G | 1e-86 | -200.216402 | 3.56% | 0.34% | motif file (matrix) |
| 2 | 0.800 | C T G A G C T A C T A G A G T C C G T A A C T G C G T A G T C A T A C G C G A T T A C G | 1e-84 | -194.294441 | 4.96% | 0.84% | motif file (matrix) |
| 3 | 0.832 | C G T A G T C A A C T G G T A C C G T A A C T G C G T A C G T A | 1e-53 | -122.048479 | 14.81% | 7.84% | motif file (matrix) |
| 4 | 0.807 | T A G C A G C T A C G T A G T C A C G T A C G T G T A C A G C T G C A T C A G T C G A T A G C T G A T C | 1e-43 | -101.244638 | 3.72% | 0.95% | motif file (matrix) |
| 5 | 0.885 | A C G T A C G T A G T C A G C T A C T G A G T C A C G T A C G T A C G T | 1e-43 | -99.063389 | 3.44% | 0.83% | motif file (matrix) |
| 6 | 0.756 | G C T A A C T G A C G T A C G T A G T C A C G T A T G C G T A C | 1e-19 | -45.924543 | 9.40% | 5.84% | motif file (matrix) |
